# Developing a Tablet-Based Self-Persuasion Intervention Promoting Adolescent HPV Vaccination: Protocol for a Three-Stage Mixed-Methods Study

**DOI:** 10.2196/resprot.5092

**Published:** 2016-01-29

**Authors:** Jasmin A Tiro, Simon Craddock Lee, Emily G Marks, Donna Persaud, Celette Sugg Skinner, Richard L Street, Deborah J Wiebe, David Farrell, Wendy Pechero Bishop, Sobha Fuller, Austin S Baldwin

**Affiliations:** ^1^ Department of Clinical Sciences University of Texas Southwestern Medical Center Dallas, TX United States; ^2^ Simmons Comprehensive Cancer Center University of Texas Southwestern Medical Center Dallas, TX United States; ^3^ Parkland Health & Hospital System Dallas, TX United States; ^4^ Department of Communication Texas A&M University College Station, TX United States; ^5^ Department of Medicine Baylor College of Medicine Houston, TX United States; ^6^ Department of Psychology University of California-Merced Merced, CA United States; ^7^ People Designs Durham, NC United States; ^8^ Department of Psychology Southern Methodist University Dallas, TX United States

**Keywords:** adolescents, intervention development, HPV vaccination, self-persuasion

## Abstract

**Background:**

Human papillomavirus (HPV)-related cancers are a significant burden on the US health care system that can be prevented through adolescent HPV vaccination. Despite guidelines recommending vaccination, coverage among US adolescents is suboptimal particularly among underserved patients (uninsured, low income, racial, and ethnic minorities) seen in safety-net health care settings. Many parents are ambivalent about the vaccine and delay making a decision or talking with a provider about it. Self-persuasion—generating one’s own arguments for a health behavior—may be particularly effective for parents who are undecided or not motivated to make a vaccine decision.

**Objective:**

Through a 3-stage mixed-methods protocol, we will identify an optimal and feasible self-persuasion intervention strategy to promote adolescent HPV vaccination in safety-net clinics.

**Methods:**

In Stage 1, we will define content for a tablet-based self-persuasion app by characterizing (1) parents’ self-generated arguments through cognitive interviews conducted with parents (n=50) of patients and (2) parent-provider HPV vaccine discussions through audio recordings of clinic visits (n=50). In Stage 2, we will compare the effects of the four self-persuasion intervention conditions that vary by cognitive processing level (parents verbalize vs listen to arguments) and choice of argument topics (parents choose vs are assigned topics) on parental vaccine intentions in a 2 × 2 factorial design randomized controlled trial (n=160). This proof-of-concept trial design will identify which intervention condition is optimal by quantitatively examining basic self-persuasion mechanisms (cognitive processing and choice) and qualitatively exploring parent experiences with intervention tasks. In Stage 3, we will conduct a pilot trial (n=90) in the safety-net clinics to assess feasibility of the optimal intervention condition identified in Stage 2. We will also assess its impact on parent-provider discussions.

**Results:**

This paper describes the study protocol and activities to date. Currently, we have developed the initial prototype of the tablet app for English- and Spanish-speaking populations, and completed Stage 1 data collection.

**Conclusions:**

Our systematic collaboration between basic and applied behavioral scientists accelerates translation of promising basic psychological research into innovative interventions suitable for underserved, safety-net populations. At project’s end, we plan to have a feasible and acceptable self-persuasion intervention that can affect key cancer disparities in the United States through prevention of HPV-related cancers.

**Trial Registration:**

ClinicalTrials.gov http://clinicaltrials.gov/ct2/show/NCT02537756 and http://clinicaltrials.gov/ct2/show/NCT02535845 (Archived by WebCite at http://www.webcitation.org/6e5XcOGXz and http://www.webcitation.org/6e5XfHoic, respectively).

## Introduction

Human papillomavirus (HPV) vaccine coverage in the United States is suboptimal (3-dose coverage in 2013 was 38% and 14% for adolescent females and males, respectively), especially among the underserved (uninsured, low-income, and racial and ethnic minorities) [1]. Guidelines recommend administration of the vaccine series to adolescents [2-5], and the Healthy People 2020 goal for 3-dose coverage is 80% [6]. However, many parents are ambivalent and often remain undecided even following a provider recommendation [7-10]. As a result, parents delay or defer making a vaccine decision [8,11]. Undecided parents are a heterogeneous group—some perceive low risk or poor vaccine efficacy, whereas others are concerned about promoting sexual behaviors, unknown side effects, or are simply not motivated [7,9-11]. Most parent-targeted interventions to date focused on reminding parents about adolescent immunizations [12-15] while few have targeted undecided parents and tried to change factors that affect decision making [16,17]. This National Cancer Institute-funded research protocol uses basic and applied social science research to develop a feasible HPV vaccine intervention, based on the principles of self-persuasion, addressing parental motivation and indecision.

Self-persuasion, defined as the process of generating one’s own arguments for changing behavior, is an effective approach to influence motivation and behavior. Based in theories of persuasion [18,19] and cognitive dissonance [20,21], basic behavioral research has demonstrated that self-generated arguments are more effective than arguments from an external source [19,21]. Approaches eliciting self-persuasion have improved diverse behaviors including smoking cessation [22], dietary behaviors [23,24], and safer sex practices [20]. Effects of self-persuasion have been shown to persist from 2-3 months [20,23] to 2-3 years [24,25]. Some argue that self-persuasion is the most effective way to change behavior because motivation for change comes from within the individual [21]. Yet, evidence is unclear about the underlying mechanisms that explain *why* self-persuasion is effective. This is particularly true among underserved populations seen in safety-net systems, given that studies to date have been conducted among diverse, but largely well-educated populations [20,22-24].

Generating one’s own arguments for changing behavior may be characterized by two processes—choice and deep cognitive processing. First, people choose which arguments, among various alternatives, are most compelling to them. Consistent with the Self-Determination Theory [26], choice elicits motivation for behavior. Across different behaviors, people are more likely to change a behavior when it has been freely chosen [27,28]. Second, people cognitively process self-generated argument content deeply [29]. Consistent with theories of persuasion [18], argument content is more likely to be convincing when processed deeply [19], because it is more accessible in memory [30]. Therefore, we hypothesize that self-persuasion will motivate parents to opt for HPV vaccination *because* they (1) choose arguments that resonate with them, and/or (2) cognitively process the arguments deeply. Examining choice and deep cognitive processing as basic mechanisms of self-persuasion is a novel synthesis of 2 research literatures. Our research protocol uses quantitative and qualitative methods to clarify each mechanism’s effects, jointly addressing an important basic science question and how to construct an optimal self-persuasion intervention for underserved populations.

By leveraging people’s own arguments for HPV vaccination, self-persuasion may be a more efficient way to deliver personally relevant messages than tailoring or motivational interviewing. Self-generated messages are similar to tailored messages [22], in which experts collect data from each patient to generate customized feedback addressing their unique needs. Tailored messages are effective *because* they are perceived as more personally relevant [31]. However, tailored interventions are time and cost intensive [32]; thus, directing parents to *generate their own* arguments for the vaccine may be a more efficient delivery method. This may be especially true for HPV vaccine decision making because determinants vary across different racial/ethnic groups [33-35]. Similarly, in motivational interviewing, an established clinical approach, providers encourage patients to verbalize arguments for changing their behavior (ie, “patient change talk”) [36,37]. Self-persuasion requires fewer trained staff, less time to complete, and may be easier to implement than motivational interviewing. Although some studies applying self-persuasion have asked people to *write* their arguments for the target health behavior [20,22,24], others have had people *verbalize* their arguments [20,24]. We hypothesize that *verbalizing* arguments using a tablet-based app will be an effective and feasible strategy for underserved populations attending safety-net clinics.

A tablet-based, self-persuasion intervention may also be valuable in priming parents to engage in vaccine discussion with their child’s provider. This approach may actually prompt parents to generate concerns or arguments *against* the vaccine—a potential negative effect [22]. However, the process of identifying concerns may also help prepare parents to express and discuss their concerns with the provider [38,39]. By timing the delivery of the tablet-based intervention immediately before seeing a provider, we can examine whether parents are more likely to respond to the provider’s cue about the vaccine and whether providers are able to address concerns. Encouraging parent-provider communication is valuable because providers are seen as credible sources of information about immunizations, particularly for underserved populations [38].

**Figure 1:**
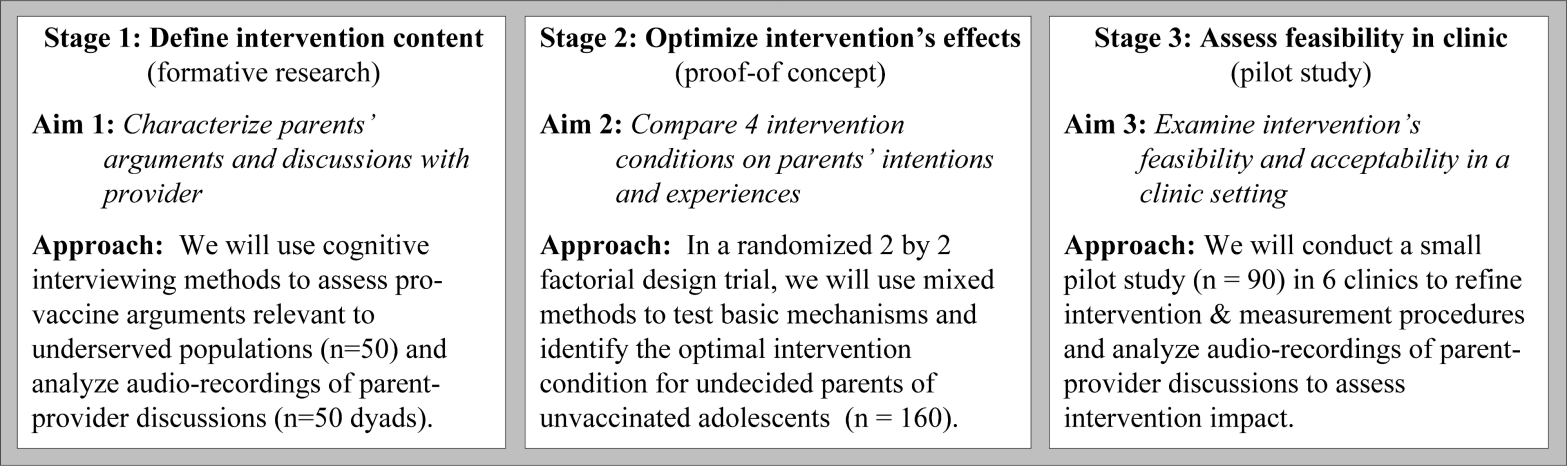
Three-stage strategy for developing and refining a parent-targeted self-persuasion intervention on adolescent HPV vaccination.

In a 3-stage strategy (Figure 1), we are using quantitative and qualitative methods to develop a tablet-based self-persuasion intervention for parents who are undecided about the HPV vaccine and test basic self-persuasion mechanisms through the following aims.

This project innovatively (1) translates basic science findings about self-persuasion into a novel intervention approach to motivate underserved parents to vaccinate their adolescents; (2) elucidates self-persuasion mechanisms that advance basic behavioral science; (3) identifies a more efficient way to elicit similar behavior change effects as tailoring and motivational interviewing; (4) characterizes the communicative environment in which HPV vaccine discussions between parents and providers occur; and (5) uses quantitative and qualitative methods to develop and refine the self-persuasion intervention approach. Our user-centered mixed-methods design synthesizes perspectives from English- and Spanish-speaking families receiving care at safety-net clinics and increases the likelihood that parents will perceive the intervention as relevant [40]. Our systematic collaboration between basic and applied behavioral scientists accelerates translation of promising basic research into innovative interventions suitable for underserved, safety-net populations. At the project’s end, we will have a feasible and acceptable self-persuasion intervention that can affect key cancer disparities in the United States through prevention of HPV-related cancers. This paper describes the study protocol and data collection activities to date (currently completed Stage 1).

## Methods

### Design

We are using a 3-stage mixed-methods design to develop and refine a tablet-based self-persuasion intervention for parents who are undecided about the HPV vaccine (Figure 1).

### Study Setting

All project activities are being conducted with patients and providers in the Parkland Health & Hospital System. As the integrated safety-net system for Dallas County, one of the largest and ethnically diverse counties in the United States, Parkland’s mission is to care for underserved, uninsured Dallas residents. Following the recommendations of the Institute of Medicine, Parkland located 21 school- and neighborhood-based pediatric clinics where there are high numbers of poor and uninsured/underinsured children [41]. Over 14,000 adolescents aged 11-17 years (68% Hispanic, 28% Black, 4% white/other) receive primary care through this system. Parkland’s HPV vaccine coverage continues to be lower than national estimates [42,43], but is consistent with clinics serving uninsured, poor populations [44,45]. If this low-rate trend continues, existing HPV-related cancer disparities may widen. Parkland has a standing order immunization policy where providers and nurses recommend all vaccines endorsed by the Advisory Committee on Immunization Practices to unvaccinated patients at all visits. Providers use a comprehensive electronic medical record (EMR) with discrete fields documenting parent refusal and vaccines administered. Parkland participates in the *Vaccines for Children* (VFC) program providing vaccines at free or reduced cost. Thus, this project’s parent-targeted self-persuasion intervention complements Parkland’s existing infrastructure. We selected the 3 neighborhood- and 3 school-based clinics with the largest volume of adolescent patients aged 11-17 years.

### Study Population

For all three stages, eligibility criteria are ascertained by EMR audit. Eligible participants are undecided parents of 11-17-year-old patients who have not started the HPV vaccine series. We specifically focus on undecided parents because they are a large population amenable to self-persuasion effects. Further, our preliminary work with parents decided against HPV vaccine administration suggests that interventions must address their specific worries and concerns. Age is restricted based on guidelines [2-5], eligibility for the VFC program, and parental consent being required for vaccine administration. We are excluding pregnant adolescents due to contraindication and parents who do not provide informed consent, lack telephone access, or have impaired hearing or speech (ie, cannot complete study activities). We ascertain parental indecision for the vaccine during recruitment. Parents who participate in Stage 1 or 2 will be excluded from later stages. For Stage 1b (audio recordings of parent-provider discussions) and Stage 3 (pilot study), we only select patients with upcoming clinic appointments.

### Recruitment

Staff receives weekly EMR reports identifying unvaccinated adolescent patients. Patient information includes name, address, telephone number, birth date, race/ethnicity, language preference, immunization history, and appointment time. Parents are mailed an invitation letter on Parkland letterhead requesting participation in a “project to improve patient satisfaction with health care and delivery of immunizations.” The letter provides a telephone number parents can use to opt-out or ask questions. Letters are sent in English and Spanish.

A few days after the mailing, parents who have not refused contact are called by a bilingual research assistant (RA) who explains the project, verifies eligibility, obtains verbal consent, and permission to review their child’s EMR, and arranges an in-person study appointment at our research offices or their Parkland clinic. To ascertain eligibility, RAs ask (1) if the child has ever had the HPV vaccine, and (2) what best describes their thoughts about it (“never thought,” “undecided,” “do not want,” or “do want”). Parents who are undecided or never thought about the vaccine are invited and consented. RAs use a computerized database to administer the baseline survey via telephone 5-14 days before the in-person appointment. For parents recruited to Stages 2 or 3, parents are randomized after completion of the baseline survey. Psychosocial variables are assessed again in exit surveys at the end of the study visit to determine changes from baseline. While we do not exclude fathers during recruitment, we expect most participants to be mothers and primary analyses are powered to analyze data from mothers. For Stages 1-3, parents will be given a US $5 gift card for completing the baseline survey and a US $20 gift card after the study visit.

### Baseline Survey

On the baseline survey, we assess parent demographics and constructs from health behavior theories or the empiric studies that have demonstrated associations with HPV vaccination behavior. Items and scales were adapted from the published literature and, if not already available, they were translated into Spanish using a multistep process [46,47]. Table 1 describes all baseline measures, estimates of their internal consistency from past studies, and whether they are also assessed during the in-person study appointment for any of the stages. Adolescent demographics, HPV vaccine behavior (date, number of doses, formula), and parental HPV vaccine decision making (acceptance or refusal) are measured via discrete fields in the EMR.

**Table 1:** Constructs measured at baseline and during study appointments.

Constructs measured at baseline (Cited studies describe survey items and psychometric properties)	Number of Items	Cronbach alpha	Measured during study appointment? (Yes, Stage(s)/No)
Parent demographics: age, race/ethnicity, sex, education, number of children	6	Not applicable	No
General attitudes toward vaccine [48,49]	5		No
Vaccine hesitancy [50,51]	10	.74-.84	No
Knowledge about human papillomavirus (HPV) disease and HPV vaccine [52,53]	7	.70	No
Intentions [54]	3	.96	Yes, Stages 1-3
Precaution Adoption Process Model decision stage [55]	1	Not applicable	Yes, Stages 1-3
Perceived susceptibility [54]	3	.94	Yes, Stages 1-3
Perceived severity [54]	3	.91	Yes, Stages 1-3
Self-efficacy [54]	2	.85	Yes, Stages 1-3
Subjective norms [11,54]	8	.78	No
Perceived benefits [48,56,57]	8	.88	Yes, Stages 1-3
Perceived barriers [54,58-60]	6	.91	Yes, Stages 1-3
Motivation for vaccination [61]	8	.73-.93	Yes, Stages 1a & 2
Trust in provider [62]	1	.86	No
Patient involvement in medical care [63]	18	.83	Yes, Stages 1b & 3

### Data Collection Approaches for each Stage

#### Stage 1

Stage 1 has two components. The goal of Stage 1a is to conduct formative research defining the tablet app’s content and creating the four self-persuasion intervention conditions that will be tested in Stage 2. For Stage 1b, the goal is to characterize parent-provider HPV vaccine discussions (Stage 1b).

##### Stage 1a

**Figure 2:**
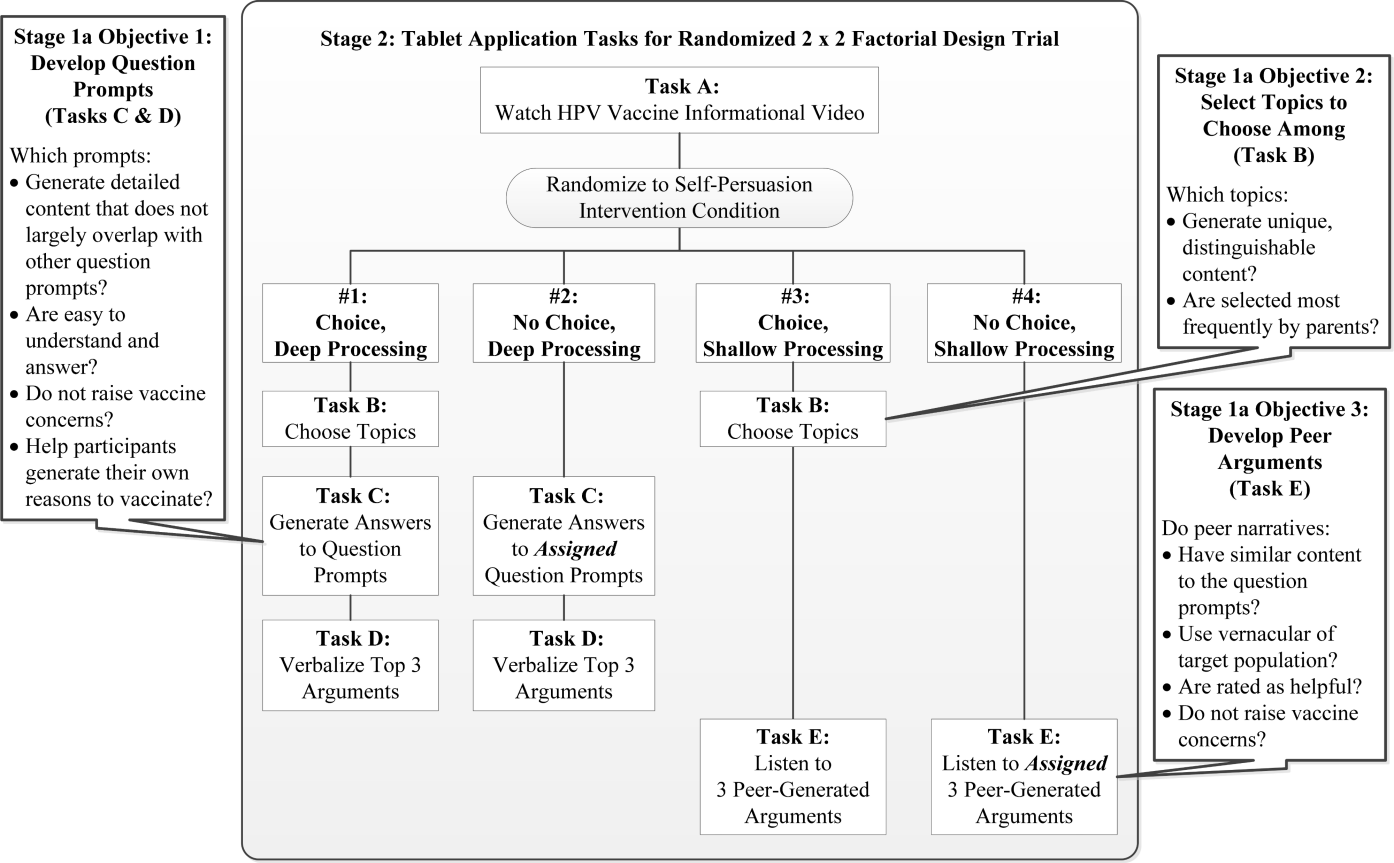
Stage 1a objectives and Stage 2 trial design with tasks parents complete while using the tablet application.

We will use cognitive interviewing methodology [64,65] to accomplish the following objectives: (1) develop and refine question prompts eliciting self-generated arguments, (2) select topics that parents can chose among, and (3) develop peer arguments (Figure 2). We will recruit parents of unvaccinated and vaccinated adolescents (ratio 1:4; *N*=50) to gather the full range of arguments for HPV vaccination. Recruitment will be also stratified on sex of the adolescent.

During the in-person study appointment, the RA will obtain consent and then show the parent how to use the tablet-based app. Voice-over instructions in English or Spanish will ensure literacy level does not inhibit parents’ understanding of information presented and tasks of the tablet app.

**Figure 3:**
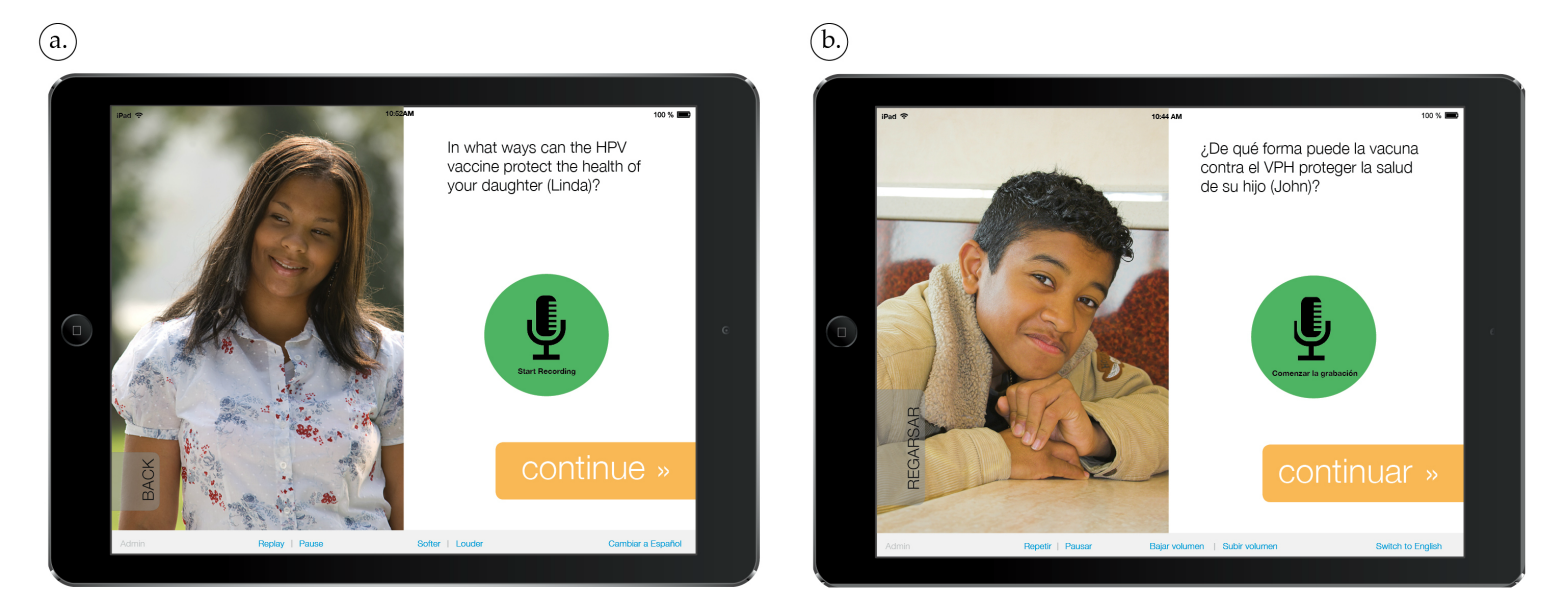
Screenshots of tablet app in (a) English and (b) Spanish.

First, the tablet will guide parents through the deep processing self-persuasion components (Tasks A, C, and D detailed in Figure 2; screenshots of tablet app are shown in Figure 3 with additional examples in Multimedia Appendix 1). In Task A, a short video provides information about HPV infection, related cancers, and the vaccine recommended for both adolescent females and males. In Task C, the tablet audio records parents as they answer a series of assigned question prompts to verbalize pro-vaccine arguments (eg, “In what ways can the HPV vaccine protect your child’s health?” or “Some parents mention concerns about the HPV vaccine. What are things doctors or other parents can say to lower parents’ concerns?”). Afterward, parents summarize their 3 most important reasons to get the vaccine for their child (Task D). Parents can play back and edit recorded responses until satisfied with them. The RA will use a cognitive interviewing-based guide to probe on comprehension of words, phrases, and vernacular. The RA will mirror participants’ own responses one-by-one and ask them to use a Likert scale to rate each question prompt for its difficulty to generate (*not hard at all* to *very hard*) and helpfulness (*not helpful at all* to *very helpful*), as well as open-ended questions to assess preferred prompts (eg, “Which were most clear?”/“Which did you dislike?”).

Then, RAs will give the tablet back, ask parents to choose among different HPV topics (Figure 2, Task B), and listen to peer-generated arguments presented in narrative format (Task E). The RA will use an interview guide to assess (1) whether parents can distinguish among the topics, (2) which topics are selected most frequently, (3) whether peer-generated arguments are clear and understandable (eg, “In your own words, what is the main point of this message?”), and (4) if arguments are helpful or raise vaccine concerns.

###### Analysis Plan for Stage 1a

Cognitive interview data will be transcribed and analyzed through techniques outlined by Willis [64]. We will develop a scheme to code participants’ responses to tablet app tasks. Through an iterative process, we will use the codes and participant ratings to examine self- and peer-generated arguments for (1) which question prompts are difficult to answer, (2) which prompts help parents generate their own arguments, (3) distribution of time spent verbalizing each response, (4) distinctions among argument topics, and (5) which argument topics are the most helpful. We will examine data by adolescent sex to ensure we select prompts and topics for Stage 2 relevant for boys and girls.

##### Stage 1b

The goal is to describe how providers convey HPV vaccine recommendations, how parents express vaccine concerns, and parents’ reactions to information from providers. We will identify parents of unvaccinated adolescents with upcoming appointments at the 3 neighborhood and 3 school clinics that see the most adolescents. After using the recruitment strategy described earlier, an RA will meet parents 15 minutes before the clinic appointment to confirm parental consent and obtain the adolescent patient’s verbal assent. The RA will place audio-recording equipment in the clinic room. After the participant meets with the provider and is discharged, the RA will conduct a 20-minute exit interview. Parents will be asked open-ended questions about their prior experience with this provider and whether the HPV vaccine was discussed during the visit. Then, the RA will administer a survey to assess household demographics, acculturation, provider recommendation, change in HPV vaccine constructs, and perceived involvement in medical care (Table 1). At least two parent-child dyads per provider will be recruited.

###### Analysis Plan for Stage 1b

Audio recordings and interviews will be transcribed and analyzed quantitatively and qualitatively.

Quantitative Analysis

We will modify Street’s Active Patient Participation Coding scheme, a well-validated observational tool for behavior coding, to analyze the audio recordings [66-68]. For parents, three types of *active* communication will be coded by trained RAs—(1) asking questions, (2) assertive expressions (offering preferences, making a request), and (3) expressing concerns (worries, seeking reassurance). Active “verbalizations” are those that influence discussion content and provider’s beliefs and behaviors [68]. Summary scores of the total number in each category per patient per interaction will be generated. Statements to both nurses and physicians are counted, as nurses are often involved in vaccine discussions [69]. For providers, we are assessing (1) vaccine recommendations; (2) rationale for their recommendation including provision of information about benefits and risks; (3) partnership building (open-ended questions encouraging patients to share opinions, feelings, ask questions, and participate in decision making); and (4) supportive talk (verbal statements of reassurance, empathy, or sensitivity) [67].

RAs will co-code 5 recordings to establish rating agreement, code 3 more semi-independently, and discuss coding as a group to resolve differences. To evaluate intercoder reliability, we will use Krippendorff alpha, a measure of agreement that allows for the analysis of categorical and continuous variables in the presence of missing data [70]. We will compute means, standard deviations, and ranges of parent and provider communications. To explore the effect of provider discussions, we will compare parents’ responses with baseline and exit survey items (Table 1) regarding the Precaution Adoption Process Model stage of decision making [55], and postvisit perceived involvement in care [63].

Qualitative Analysis

We will perform in-depth thematic analysis of all transcripts using NVivo 9.0. Through iterative coding and interpretation within and across transcripts, a team of bilingual staff trained in qualitative methods will code actual utterances, expressions, and concepts against participant characteristics to identify themes and relationships [71]. We will organize these codes into a codebook that relates data to behavioral theory [72]. Regular meetings will enable the team to test emergent themes and interpretation against the knowledge base of experts in pediatrics, self-persuasion, vaccination, and patient-provider communication.

At the end of Stage 1, we will know (1) the range of provaccine arguments underserved parents generate, (2) which arguments are easiest to generate and most prevalent, (3) which peer-generated arguments are rated as clear, comprehensible, and distinct from other arguments, and (4) range and degree to which parents participate in HPV vaccine discussions. With these data, we will select the optimal argument topics for the self-persuasion intervention conditions. Baseline descriptive information about the communicative environment will be compared with parent-provider discussions after exposure to the optimal self-persuasion intervention in Stage 3.

#### Stage 2

Stage 2 is a proof-of-concept randomized controlled trial (RCT) in which we will randomly assign 160 undecided parents to one of the four intervention conditions using a 2 (argument topic choice: parents choose vs parents are assigned topics) × 2 (cognitive processing level: parents verbalize vs parents listen to arguments) factorial design (Figure 2). Based on randomization status, the tablet app directs parents to either verbalize their own arguments based on topics they choose (Condition Number 1: deep processing, choice), verbalize arguments based on topics assigned to them (Condition Number 2: deep processing, no choice), listen to arguments based on topics they choose (Condition Number 3: shallow processing, choice), or listen to arguments based on topics assigned to them (Condition Number 4: shallow processing, no choice). Verbally generating (vs reading) material is known to elicit deep cognitive processing [73]. Offering people choice among alternatives has been used to elicit intrinsic motivation for the target behavior [74]. We will use a quantitative approach to test for changes in parents’ HPV vaccine intentions and a qualitative approach to compare parents’ experiences with intervention tasks to determine which intervention condition is optimal to elicit self-persuasion and minimize negative reactions in our underserved population.

##### Hypotheses

- Changes in intentions will be higher for deep processing (Conditions 1 and 2) compared with shallow (Conditions 3 and 4) and choice (Conditions 1 and 3) compared with no choice/assigned (Conditions 2 and 4). We are testing two main effects.
- Parents in Conditions 1, 2, and 3 will report experiences with intervention tasks that differ on (1) likeability, (2) usefulness, (3) difficulty, and (4) relevance to discussion with child’s provider.

We will identify undecided parents using the same recruitment procedures described above and stratify recruitment based on adolescent sex (80 girls and 80 boys). During a 1-hour study appointment at the child’s clinic, parents will complete tablet app tasks depending on randomization status (Figure 2). After using the tablet, an RA will conduct an exit interview to assess parents’ perspective on the tablet app, self-persuasion condition, and how their beliefs and experiences shape feelings about the HPV vaccine. The RA will audio record parent responses to the following topics:

- *Quantitative outcomes:* For our *primary outcomes*, we will reassess parents’ HPV vaccine intentions and decisional stage. As a *secondary outcome*, we will reassess parents’ perceived benefits and barriers. To assess change, we will compare responses to the baseline survey. Using Likert scales, we will ask about parents’ experience using the tablet app with respect to (1) likeability, (2) usefulness, (3) difficulty, and (4) relevance for a discussion with their child’s provider. Adolescents who accompany their parents to the study appointment can receive the first dose from a Parkland nurse immediately after the exit interview. The nurse will record this dose in the adolescent’s EMR to ensure that s/he can complete the series through the VFC program. We will use the EMR to assess administration of all HPV vaccine doses.
- *Qualitative process outcomes:* To determine which conditions are optimal for our underserved population, RAs will observe parents as they use the tablet app and will use open-ended questions to evaluate whether the process raised new vaccine concerns [negative outcome] or addressed concerns [positive outcome].
- *Manipulation checks of choice and cognitive processing*: These checks will provide additional evidence for the hypothesized processes of self-persuasion and help inform which condition we will test in Stage 3.

- *Motivation:* As *a manipulation check of choice*, we will assess motivation for vaccination with a modified Treatment Self-Regulation Questionnaire [61].
- *Memory:* As *a manipulation check of deep cognitive processing*, we will ask parents at the end of the interview to recall as much as they can from the arguments they verbalized/heard [75]. Independent raters will code parents’ responses to determine memory accuracy.

##### Quantitative Analysis

Across self-persuasion conditions, demographic characteristics will be compared at baseline. If intervention groups differ on any of these variables, further analyses will be conducted both with and without these variables as covariates to determine whether these demographic variables are of relevance to group differences. We will compare the effects of choice and cognitive processing on vaccination intentions (primary outcome) using linear regression. Independent variables will be dummy-coded variables based on two main effects (choice: high=0, low=1; processing: deep=0; shallow=1), plus their interaction. If equivalence assumptions of initial scores and parallel regression slopes for the groups are met, baseline intentions will be included as a covariate to properly model change [76]. If not, repeated measures analysis of variance will be used [77]. We anticipate changes in intentions will be highest in the deep processing, choice condition, indicating an additive effect. We will also explore the interaction of the two effects.

##### Qualitative Data Analysis

We will use the same analytic process described for Stage 1b.

##### Sample Size

We powered this proof-of-concept trial to test hypothesized effects on a surrogate marker, HPV vaccine intentions. It was determined by possible effect sizes (*f*_2_) for each main effect, the number of predictor variables for each effect, and the total number of independent variables and covariates in the model (ie, 4, namely, pretest vaccine intention, two main effects, and the interaction). To detect an effect size of *f*_2_=.05, between a small (*f*_2_=.02) and medium effect (*f*_2_=.15), with 80% power and a 5% Type I error rate, we need 160 participants (40 per condition). The sample size will also be sufficient to achieve saturation needed to observe the range of qualitative outcomes.

##### Synthesis of Quantitative and Qualitative Analyses

To determine which self-persuasion condition (1, 2, or 3) is optimal for our safety-net population and which condition will be tested in Stage 3, we will triangulate quantitative and qualitative findings by creating a summary profile for each condition. The optimal condition will be one that has a positive effect on intentions, but also minimizes participants’ negative reactions to using the tablet app. A condition that does not affect intentions will not be considered optimal, regardless of its effect on other quantitative and qualitative outcomes. Likewise, a condition that affects intentions but for many participants raises new concerns, is rated as difficult to complete, or takes significant time to complete will not be considered optimal.

At the end of Stage 2, we will have quantitative and qualitative data that clarify whether it is best to ask parents to verbalize their own arguments, to choose argument topics they prefer, or both. Evidence clarifying which of the two specific mechanisms (deep processing and choice) has an effect, or whether they have an additive effect, will be critical to how we select the optimal self-persuasion condition to implement in Stage 3. For example, if there is an effect of processing but not choice, we would use Condition 2 that has parents verbalize arguments based on assigned topics that are most persuasive rather than allowing them to generate arguments based on chosen topics that may be less persuasive. If there is an effect of choice but not processing, we would use Condition 3 that has parents choose argument topics they want to hear rather than having them go through the more taxing process of verbalizing their own. If both have an effect, Condition 1 will be selected for Stage 3. Given these possibilities of different intervention approaches, our mixed-methods approach maximizes our ability to identify the most optimal intervention condition. Findings will also inform basic behavioral research by generating evidence for specific mechanisms of self-persuasion.

#### Stage 3

In Stage 3, we will assess feasibility of implementing the optimal intervention, identified in Stage 2, through a pilot RCT with 90 parents in 3 neighborhood and 3 school clinics. Parents will be randomly assigned to either (1) self-persuasion plus information (specific operationalization will be determined in Stage 2; n=45) or (2) HPV information only (n=45). Parents will be asked to come 30 minutes prior to the clinic appointment to meet the RA. For the self-persuasion group, we will follow procedures outlined in Stage 2. The tablet will play the educational video (Figure 2, Task A) to parents in the information-only group. Based on procedures used for Stage 1b, we will audio record the parent-provider discussion. Immediately after the visit, an RA will conduct an exit interview in which participants will be asked questions about whether the tablet app was useful, relevant, culturally appropriate, if they had sufficient time to complete intervention procedures, and their communication with the provider.

#### Outcomes and Analyses

We designed this pilot RCT to obtain feasibility information on recruitment, clinic implementation issues, and estimation of intervention effects that are key for developing a subsequent efficacy RCT.

##### Enrollment Rates

We will assess whether enrollment rates are similar across clinics.

##### Sufficient Time for Intervention Procedures

Because clinics may differ in their patient flow and visit wait times, we will track the number of participants who complete the tablet app within the time constraints allowed by the clinic and determine whether time allotted is similar and sufficient across all sites. We expect an 85-90% completion rate to determine feasibility.

##### Potential for Contamination

We will determine the appropriate level of randomization (patient, provider, or clinic) and the degree to which contamination occurs at each level. We will use visit history data in the EMR to examine the percentage of patients who visit more than 1 clinic and see more than 1 provider. For example, if there is significant crossover of patients to different providers at the same clinic, then we will randomize at the clinic-level in the subsequent efficacy trial.

##### Intermediate Outcome: Active Parent Participation

We hypothesize that exposure to the self-persuasion intervention will positively influence active parent participation in discussions with providers. We will apply Street’s scheme to code the 3 types of active communication (Stage 1b) [67]. To estimate effect sizes for the subsequent efficacy RCT, we will compute means, standard deviations, and ranges of parent and provider communications and compare them to data collected in Stage 1b. We will use multivariable mixed linear regression modeling to explore factors associated with parent degree of participation (eg, English vs Spanish language) [78]. This method models the provider as a random effect to adjust for potential clustering of patients by provider; parent/patient characteristics of interest will be modeled as fixed effects.

##### Primary, Quantitative Outcome: HPV Vaccine Uptake

We hypothesize that exposure to the self-persuasion intervention will increase 1-dose and 3-dose HPV vaccine coverage rates. We will use the EMR to measure vaccine uptake. These data will help estimate intervention effect sizes of the self-persuasion intervention, compared with the information-only group, guiding the design and sample size for the subsequent efficacy RCT. We will also measure HPV vaccine-specific measures of intentions, benefits, and barriers (Table 1).

#### Sample Size

To estimate the sample size necessary to establish feasibility, we used a confidence interval approach and formula for obtaining a 95% CI for a single proportion. Assuming a priori criterion of success if 1-dose coverage is 70% or more of eligible adolescents and a margin of error of 0.05, the required sample size would be at least 90 patients.

After completing Stage 3, we will have quantitative and qualitative data to determine whether our self-persuasion intervention is feasible and acceptable across clinics—data that will guide us in refining intervention and measurement procedures. Thus, at the end of this stage, we will have a well-characterized and feasible intervention promoting HPV vaccination ready to be tested in future efficacy trial.

## Results

### Initial Prototype of the App

To date, we have developed the initial prototype of the tablet app and completed Stage 1. Here we summarize the following aspects of the tablet app design to ensure cultural appropriateness for our diverse, low-literacy study population: (1) content of the educational video, (2) conceptual equivalence of content for English- and Spanish-speaking parents, and (3) relevance and appeal to parents of adolescent boys and girls.

### Educational Video Content

Educational content was derived from published sources and previously tested educational materials adapted to a 6th grade reading level for low literacy populations [79,80]. We designed messages to address constructs (perceived risk, perceived benefits, perceived barriers [safety and side effects], and anticipated regret) important to our safety-net population based on our formative research and the empiric literature [43,81-83]. The goal of the video was to provide basic vaccine-related facts so that all parents would have the same baseline knowledge of the HPV vaccine prior to completing self-persuasion intervention tasks (Figure 2, Task A). The educational video content was written by co-investigators, translated into Spanish through a multistep process by a bilingual committee (detailed in the following section), and reviewed by our community advisory board (CAB).

We convened 3 CAB meetings during development of the educational video, 2 in English and 1 in Spanish. The sixteen CAB members included social workers who specialize in medical, immigration, and children’s services; parents of adolescents; clinic administrators and medical staff; outreach workers; health educators and translators; and community program directors. Each meeting was conducted with at least two research staff to facilitate the discussion and take notes. CAB members each used the iPad independently at the beginning of the meeting to enable detailed feedback and discussion. Members suggested changes to the content and format of the educational video, including facts about the sexual activities that lead to HPV transmission, neutral (nongraphic) images of HPV and its effects on the human body, the pace of the voice-over narration, and text font size. Members at the Spanish CAB meeting stressed the importance of maintaining community trust by giving parents unbiased facts and suggested language to increase parents awareness of what the vaccine does and does not protect against.

### Conceptual Equivalence of Study Materials for English- and Spanish-Speaking Parents

All materials (invitation letter, surveys, and tablet app content) went through a multistep translation process in which materials were translated into Spanish, back-translated, tested using cognitive interviewing methods with the target population, and reviewed by a bilingual committee representing several Latin American countries [46,65]. The goal of the translation process was to create conceptually equivalent materials for both English and Spanish speakers and to strive for “broadcast” Spanish (eg, understandable to immigrants from all Spanish-speaking countries) [47,84,85]. The 6-member committee identified potentially problematic concepts (eg, higher literacy phrases and grammar). They designed a cognitive interviewing guide to probe for problems with comprehension and cultural appropriateness.

To accomplish this goal, the committee had to decide when words and phrases should differ between the English and Spanish versions. For example, the phrase “It eases my mind to know the vaccine was carefully tested” was translated as “Me tranquiliza (feel calm) saber que la vacuna fue cuidadosamente probada” to facilitate comprehension of the emotion. In addition, when translating the phrase “better prevent now, than regret later” into Spanish, the words “now” and “later” were dropped to use a well-known phrase in Spanish—“Más vale prevenir que lamenter (ie, better to prevent than to lament).” After cognitive testing in both languages, the committee sometimes identified that the best solution was to change the English text. For example, “chance/oportunidad” was systematically changed to “risk/riesgo.” The concept “chance” is appealing from a literacy perspective; however, committee members argued that it did not fully convey the potential for an adverse consequence. Screenshots from the English and Spanish versions of the tablet app are shown in Figure 3 and Multimedia Appendix 1.

### Relevance for Parents of Adolescent Boys and Girls

Based on formative research findings in the HPV vaccine literature [82,83,86], the tablet app was targeted to the sex of the child in two ways: gender of the narrator and predominant images selected. Investigators and staff met thrice to evaluate potential male and female narrators for each language and made a final selection based on consensus. Narrators were evaluated based on accent, pitch, and pace that would appeal to parents in our geographic region. The images of the children, single-parent, and two-parent families reflected the racial and ethnic distribution of our target population (see Figure 3 and Multimedia Appendix 1). While both the English and Spanish versions depicted African American, Latino, and white families, a larger proportion of African American images were selected for the English version of the app and Latino images for the Spanish version.

CAB members’ feedback on narrators and images were positive overall. Members remarked that the images were visually appealing, but requested more images of boys and a broader range of skin tones for the African American images. CAB members felt that it was important to maintain gender concordance of child and narrator for parents, and appreciated that the audio and text always matched. Bilingual CAB members were asked to test both the English and Spanish versions of the app and compare their experiences; they reported that the voice-over narration was clear in tone, had a good pace, and would be understandable to parents from any Spanish-speaking country.

## Discussion

### Vaccine Coverage in the United States

HPV vaccine coverage among US adolescents is suboptimal and interventions that address parental decision making are urgently needed. Self-persuasion—generating one’s own arguments for a health behavior—may be particularly effective for parents who are undecided or not motivated to make a vaccine decision. Through a three-stage design, we will identify an optimal and feasible self-persuasion intervention strategy to promote adolescent HPV vaccination in safety-net clinics.

There are some study design limitations that warrant mention. First, in our 2 × 2 factorial trial (Stage 2), we opted not to include 1-dose coverage as an outcome. Given that the purpose of Stage 2 is to understand basic mechanisms and refine and optimize the intervention by examining individual components of it, we opted to conduct the study in a more controlled setting than a clinic visit. As a result, unless adolescents accompany parents to study appointment (which is not required for participation) as they would to a clinic visit, we will be unable to assess vaccine uptake. Instead, we opted to assess vaccine intentions as the primary outcome because meta-analytic evidence suggests that experimentally induced changes in behavioral intentions lead to subsequent changes in behavior [87]. Moreover, we will assess 1-dose and 3-dose coverage (Stage 3), so we will have evidence for the intervention effect on vaccine behavior that will be critical for designing a future efficacy trial. Second, the studies across the three stages are not sufficiently powered to definitively examine potential race/ethnicity and sex differences in the intervention. This is important given that factors influencing parental motivation may differ depending on ethnic/cultural background and whether the child is a girl or boy [43]. However, we will be able to *explore* these potential differences in this study to generate preliminary data about variables that moderate the self-persuasion intervention’s effect and thus consider powering the future efficacy trial to test potential moderators. Third, we did not include question prompts to directly rebut vaccine concerns and we excluded parents who were decided against the HPV vaccine. In our preliminary work, we have found that prompting parents to think about vaccine concerns can raise concerns that they were not thinking about without prompting, and persuade parents against vaccination. We believe a separate intervention approach focused on addressing worry and concerns is warranted for these “decided against” parents; thus, it is best addressed in a separate study.

### Conclusions

This project’s findings will inform basic research by testing specific theoretical mechanisms underlying self-persuasion and providing evidence to support and guide future basic research in self-persuasion. It addresses underserved populations (uninsured, poor, racial, and ethnic minorities) who have high incidence and mortality from HPV-related cancers. The project will enhance the capability of safety-net clinics to promote HPV vaccination by developing a self-persuasion intervention addressing parental indecision. Our three-stage intervention development strategy takes several steps to ensure the usability and cultural appropriateness of all project materials for underserved populations. We are leveraging Parkland’s existing EMR to identify eligible patients and evaluate the intervention’s impact on HPV vaccine uptake. Our intervention approach holds promise to be institutionalized by Parkland, adapted for other cancer prevention behaviors (eg, smoking cessation, physical activity), and adopted by similar safety-net systems if shown effective in the future efficacy trial.

## Appendix

Multimedia Appendix 1 English and Spanish screenshots depict each of the tasks parents complete while using the tablet application.

## Abbreviations

CAB: community advisory board
EMR: electronic medical record
HPV: human papillomavirus
RA: research assistant
RCT: randomized controlled trial
VFC: Vaccines for Children program
