# Supplementary material for: Developing a Tablet-Based Self-Persuasion Intervention Promoting Adolescent HPV Vaccination: Protocol for a Three-Stage Mixed-Methods Study
Source: JMIR Res Protoc. 2016 Jan 29;5(1):e19. doi: 10.2196/resprot.5092 (PMC4752693; doi:10.2196/resprot.5092)
Supplement: Multimedia Appendix 1 [file resprot_v5i1e19_app1.pdf]

Multimedia Appendix 1- English and Spanish screenshots depict each of the tasks parents complete while using the tablet application.

Introduction to the Tablet Application

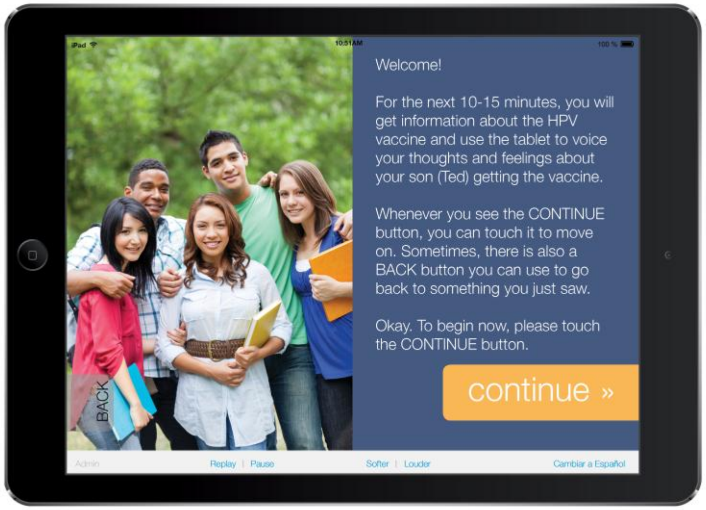

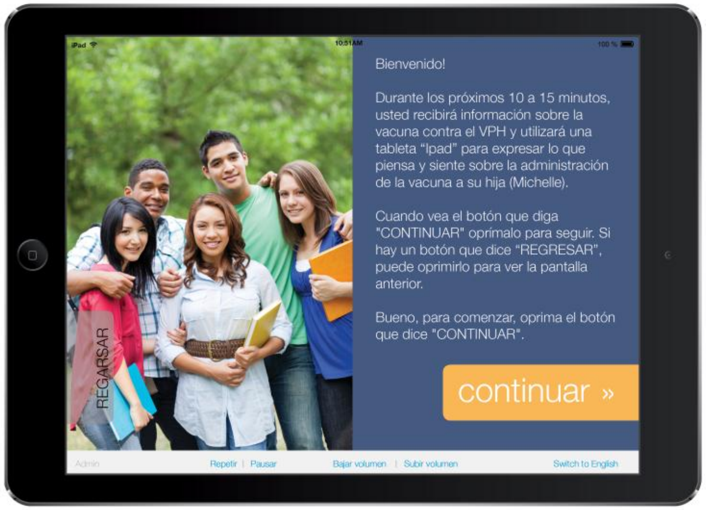

Task A: Informational Video

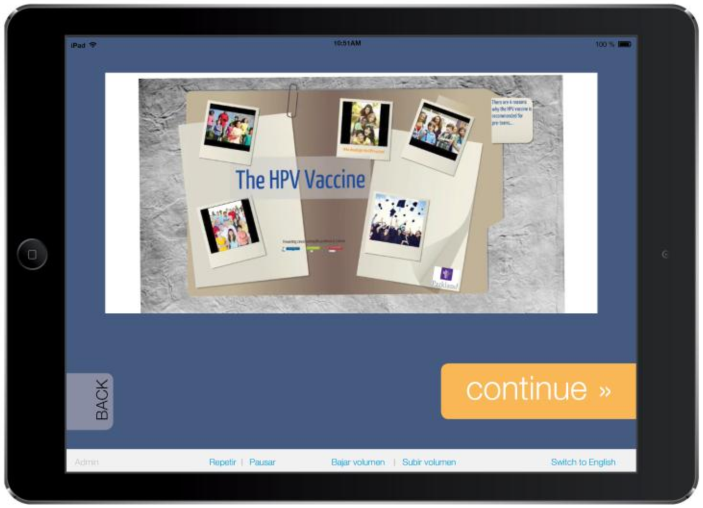

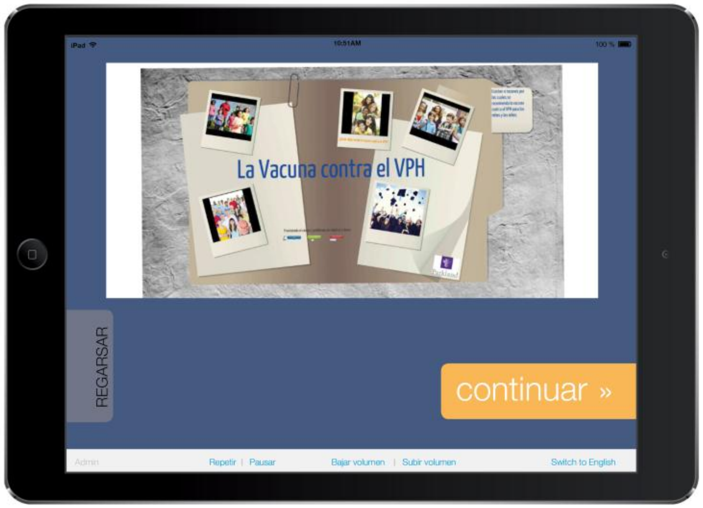

Task B- Choose Topics for Intervention Conditions #1 and #3

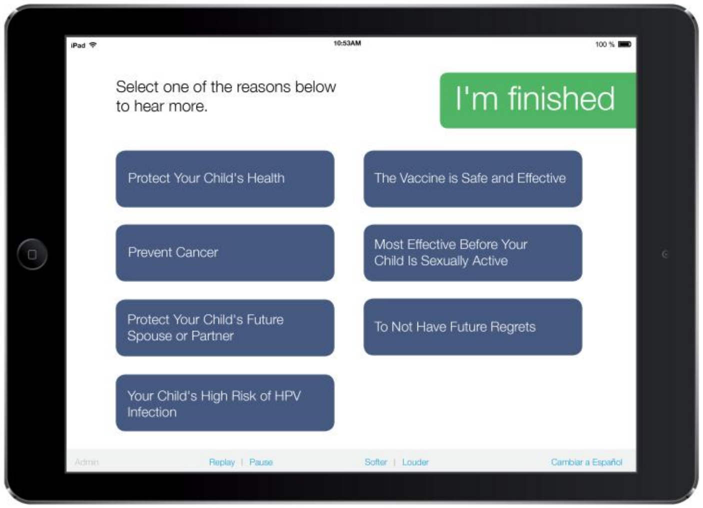

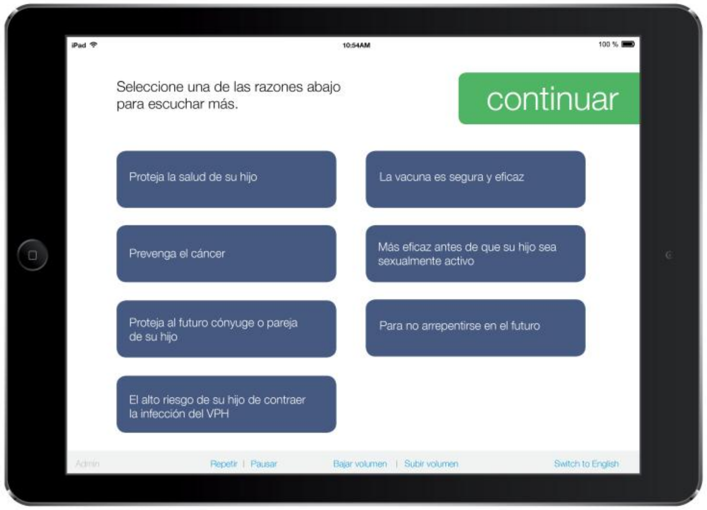

Task C – Answer Question Prompts for Intervention Conditions #1 and #2

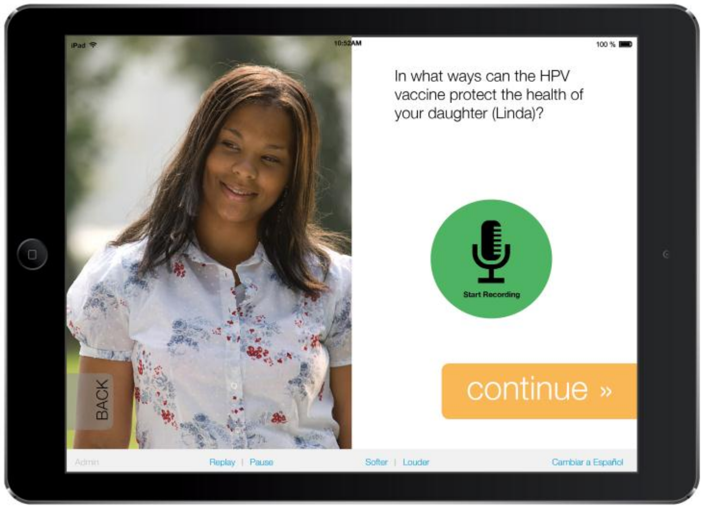

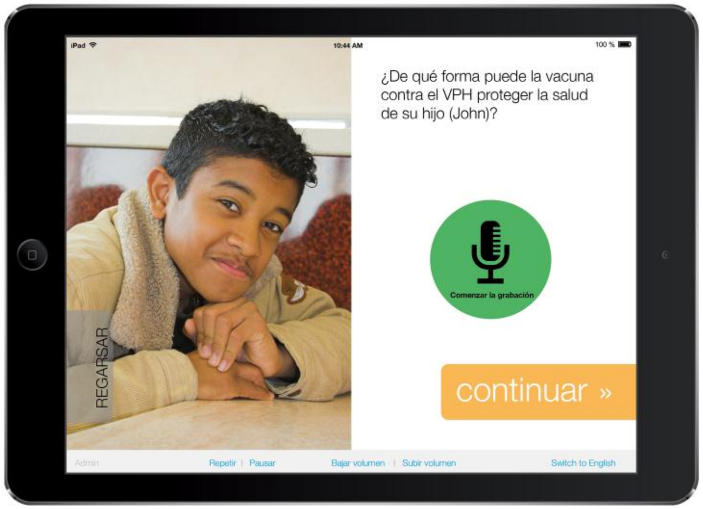

Task D – Verbalize Top 3 Arguments for Intervention Conditions #1 and #2

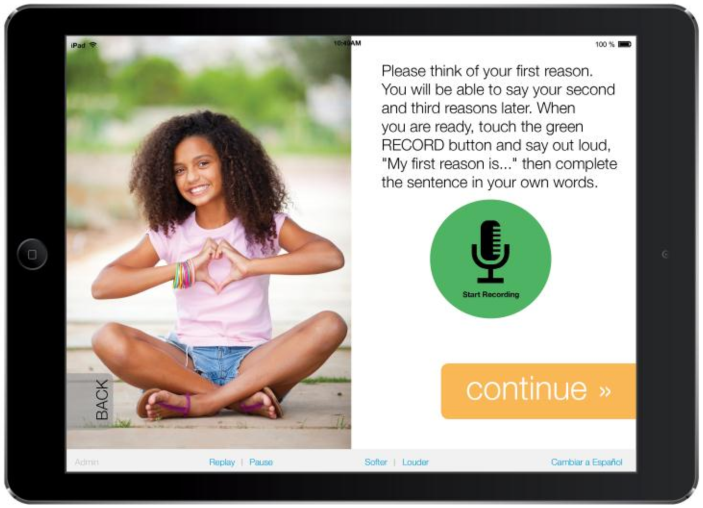

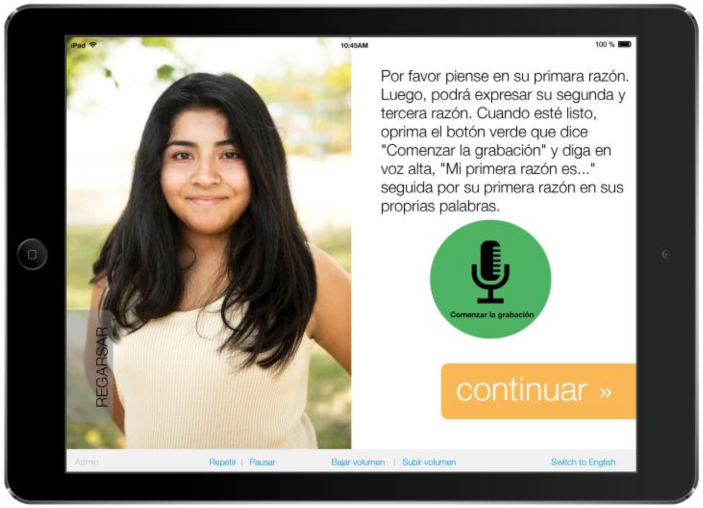

Task E – Listen to Peer Arguments for Intervention Conditions #3 and #4

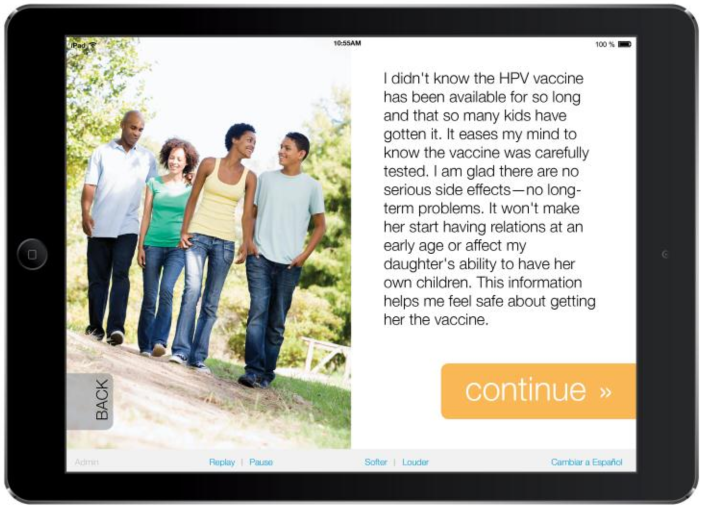

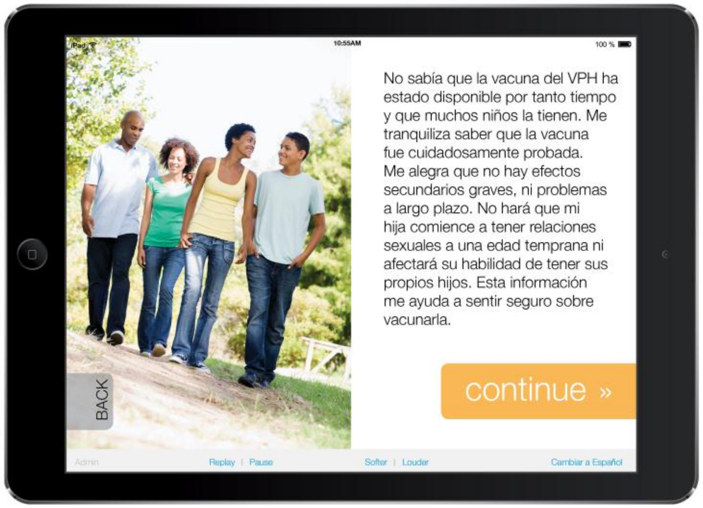

Exit Screen

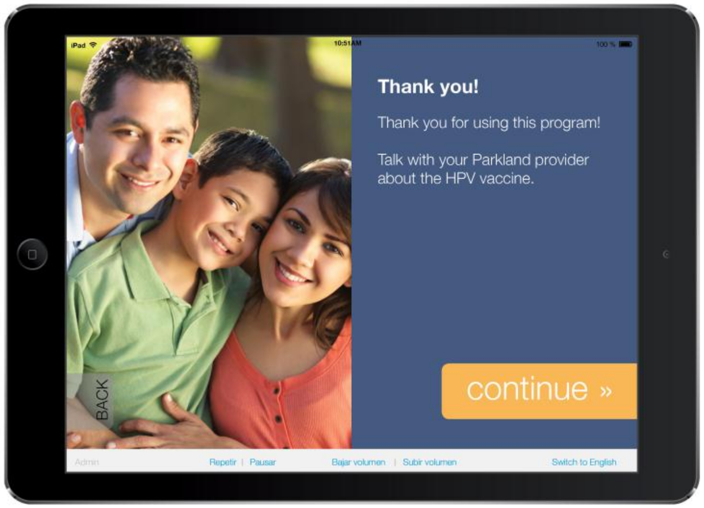
Thank you!
Thank you for using this program!
Talk with your Parkland provider about the HPV vaccine.
continue »
BACK
Admin
Repetir | Pausar
Bajar volumen | Subir volumen
Switch to English

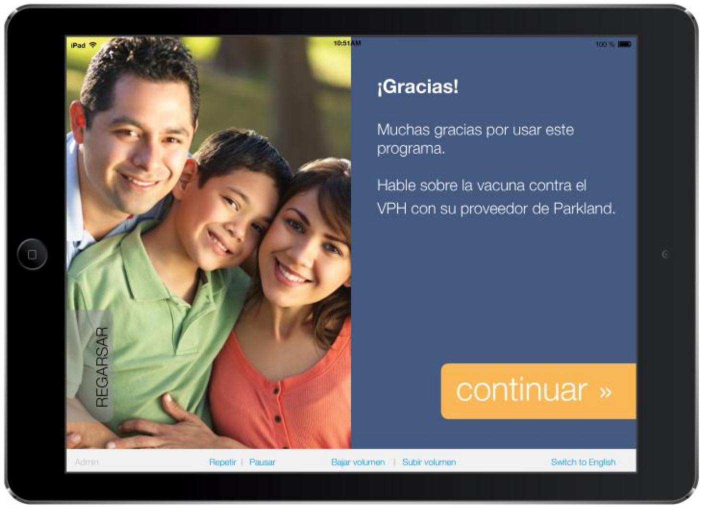
¡Gracias!
Muchas gracias por usar este programa.
Hable sobre la vacuna contra el VPH con su proveedor de Parkland.
continuar »
REGARSAR
Admin
Repetir | Pausar
Bajar volumen | Subir volumen
Switch to English
